# A high risk of postoperative periprosthetic femoral fracture in Dorr type C femurs: a retrospective cohort study with 10-year follow-up data and a preliminary monochromatic image analysis

**DOI:** 10.1097/JS9.0000000000000810

**Published:** 2023-10-11

**Authors:** Mingyang Li, Yi Zeng, Yong Nie, Kai Liao, Fuxing Pei, Jing Yang, Huiqi Xie, Bin Shen

**Affiliations:** a Department of Orthopedic Surgery and Orthopedic Research Institute, West China Hospital; b Department of Radiology, West China Hospital, West China Medical School, Sichuan University, Chengdu, Sichuan Province, People’s Republic of China

**Keywords:** bone growth, Dorr type C femurs, short stems, spectral CT

## Abstract

**Background:**

The authors applied Anatomique Benoist Girard II (ABG II) stems for total hip arthroplasty in some Dorr type C femurs as early attempts. Here, the authors compared the long-term follow-up results between ABG II stems and the ʻwell-performingʼ Corail stems and their monochromatic images.

**Methods:**

Among 3214 primary total hip arthroplasty records, 43 short ABG II stems and 67 standard-length Corail stems implanted in Dorr type C femurs were eligible and enrolled in this retrospective cohort study, with a mean follow-up of 10.3 years. Revision rates, Harris hip scores, and radiologic signs were compared. Spectral CT scans from a representative sample were obtained, and monochromatic images were reconstructed. A quantitative method was developed to measure the volume of the gap around stems. Patient-specific finite element analysis was conducted to investigate the strains.

**Results:**

The revision rate of ABG II stems was significantly higher than that of Corail stems (21 vs. 3%, *P*<0.05). In the monochromatic images, fewer spot-weld signs (2.2 vs. 3.4, *P*<0.05) and wider gaps around stems (1.64 cm^3^ vs. 0.13 cm^3^, *P*<0.05) were observed on average in the ABG II group. The mean maximum principal strains of the proximal femurs in the ABG II group were close to the yield strains and significantly larger than those in the Corail group (0.0052 vs. 0.0011, *P*<0.05).

**Conclusions:**

There was a high risk of postoperative periprosthetic femoral fracture for ABG II stems in Dorr type C femurs. Monochromatic images provided some insight into the failure mechanism.

**Level of Evidence:**

III

## Introduction

Highlights

- Several studies have demonstrated good function scores and high survival rates for short cementless stems in Dorr type C femurs in short-term to mid-term follow-ups. However, this is the first study (as far as we know) that reported long-term results. We detected an extremely high revision rate of the ABG II stems in Dorr type C femurs.
- We introduced monochronic energy images to evaluate bone growth and strains between the ABG II and the Corail stems. Those results explained the poor performance of ABG II stems in Dorr type C femurs to some extent.
- Based on the high revision rate of the ABG II stem in Dorr type C femurs reported in this study, the precious contradiction in the long-term results of ABG II stems might be partially explained by the variation of the femoral type in the included subjects.

Both standard-length and short cementless femoral stems have achieved excellent long-term survivorship in general femurs. Because the initial stability of cementless femoral stems is achieved by press-fitting slightly oversized components and durable fixation is obtained by bone growth that anchors implants firmly in femoral canals, the results in femurs with wide patulous canals and poor bone quality, which were described by Dorr as type C femurs^1^, might differ from results in general femurs.

Standard-length stems were frequently applied in Dorr type C femurs in previous studies. However, some limitations of standard-length cementless stems, such as proximal–distal mismatch^2^, more intraoperative fractures^3^, and loss of bone stock in revision, are highlighted in Dorr type C femurs, which led to our exploration of short cementless stems. Short stems offer specific advantages, including better bone preservation, avoidance of proximal–distal mismatch, lower rates of intraoperative fracture^4^, natural load transfer^5^, smaller incisions, and less stress shielding, and bone resorption^6^. Some of these advantages seem quite promising for the characteristics of Dorr type C femurs. Short-term to mid-term follow-up studies have demonstrated good functional scores and high survival rates for short cementless stems in Dorr type C femurs^7–9^. Therefore, we implanted Anatomique Benoist Girard II (ABG II) stems in some patients with Dorr type C femurs as early attempts. The ABG II stem is a typical short stem that has been successfully used in general femurs, with good fixation^10^, a high fill ratio^11^, and a survival rate over 98% at an average 10-year follow-up^12,13^. However, the survival rate, fixation, and biomechanical characteristics of ABG II stems in Dorr type C femurs are still unknown.

We implanted ABG II stems in some patients with Dorr type C femurs as early attempts; however, unfortunately, we felt there was a high risk of fracture in the series. In fact, the results of short stems in Dorr type C femurs are contradictory in previous mid-term reports, and long-term results are still lacking. Gkagkalis *et al*.^14^ reported an extremely high periprosthetic femoral fracture (PFF) rate for short stems (Optimys) in Dorr type C femurs, while Zhen *et al*.^15^ detected no revisions in their 5.5-year follow-up period. Therefore, we compared the survival rate and radiologic signs between ABG II and Corail stems (one well-performing stem in our practice) over the long-term and further investigated bone growth and proximal strains through quantitative measurements based on monochromatic spectral CT images and patient-specific finite element analysis (FEA).

## Methods

This study was divided into two parts: a retrospective cohort study including the standard follow-up; and a monochromatic image analysis of a portion of the cohort. This study has been reported in line with the strengthening the reporting of cohort, cross-sectional, and case–control studies in surgery (STROCSS) criteria^16^ (Supplemental Digital Content 1, http://links.lww.com/JS9/B179).

### Sample size estimation

The sample size was estimated according to the formula for comparing two proportions in the setting of two-sample, two-sided equality^17^. We speculated that ABG II stems have a high revision rate (22.2%) according to Gkagkalis’ study, which similarly reported the mid-term results of short stems in Dorr type C femurs^14^. The revision rate of Corail stems was estimated as 0.05%, which was a weighted average from previous reports on the long-term survival of standard-length stems in Dorr type C femurs^18–22^. The type I error rate was set as 0.05, and the type II error rate was set as 0.2. The sample size was estimated to be 32 in each group. With the rate of loss to follow-up set as 20%, 39 hips in each group were needed. The percentage of Dorr type C femurs was roughly estimated as 10%^23,24^. According to the utilization rate of ABG II stems at our institution, we estimated that screening 3000 total hip arthroplasty (THA) records would satisfy this research.

### Subjects

From January 2006 to March 2012, a total of 3048 patients underwent 3214 primary THAs performed at our center by three senior joint surgeons (P.F.X., Y.J., S.B.), each of whom performed more than 100 THAs per year. Stems were selected at the surgeon’s discretion. Patients were excluded if they had an apparent femoral deformity, a hip joint tumor, or incomplete medical records.

This study was registered in the Chinese Clinical Trial Registry (ChiCTR 1900026467). Approval for this retrospective cohort study was obtained from the institutional review board of West China Hospital (Chengdu, Sichuan Province, China) (NO 268,2012), and all included patients provided informed consent.

Referring to Dorr’s description of femurs^1^, we identified type C femurs based on preoperative plain anterior-posterior and lateral X-ray films. We also applied the quantitative criteria described by Nakaya *et al*.^25^ when the morphology used for classification was not obvious.

Both the ABG II and Corail stems are cementless, metaphyseal fixed stems with a hydroxyapatite (HA) coating. The ABG II is a short anatomic stem made of titanium, molybdenum, zirconium, and iron. At the metaphyseal level, the stem has a scale-shaped design and is coated with a 70-micron HA layer. The tail of the implant is short and ultra-polished to avoid contact with the diaphysis and fixation at this level. The Corail stem that we used is a collarless, straight, standard-length, double-tapered version. The stem is made of forged titanium alloy (TiAl6V4). A 150-micron HA coating is applied to the entire stem. ABG II stems from size 3 (110 mm in length) to size 7 (135 mm in length) and Corail stems from size 10 (140 mm in length) to size 13 (155 mm in length) were applied in our research. All ABG II stems and no Corail stems in this research satisfied the definition of a short stem (length less than twice the vertical distance from the tip of the greater trochanter to the base of the lesser trochanter^26^) (Supplementary Fig. 1, Supplemental Digital Content 2, http://links.lww.com/JS9/B180).

### Routine measures

The routine follow-ups were at 6 months and 12 months after surgery and every 2 years afterward and included an anterior-posterior and lateral hip X-ray examination and functional evaluation. The functional evaluation was based on the HHS, which includes pain, function, absence of deformity, and range of motion^27^. The mean duration of follow-up in this study was 10.3 years (range, 7.6–13.9 years).

The revision rate, degree of satisfaction, and postoperative HHSs were evaluated and recorded. With a revision of the femoral component as the clinical endpoint, survival statistics were calculated and plotted with the Kaplan–Meier method. To minimize the confounding factors, a multivariable Cox regression including age, sex, BMI, preoperative HHS, time of surgery, fill ratio at the lesser trochanter on the anterior-posterior X-ray, and diagnosis was conducted.

Stability and stress shielding were classified; spot welds, osteolysis, radiolucent lines, heterotopic ossification, and pedestals were recorded as either present or absent. The stability of the stem was evaluated according to the criteria set by Engh *et al*.^28^. Stress shielding was determined by the classification reported by Engh *et al*.^29^. Spot welds were defined as new bone formations bridging the porous stem surface and the endosteal bone. Osteolysis was defined as a radiolucency with a scalloped or cystic shape or a progressive radiolucency greater than 2 mm in width. A pedestal was defined as a shelf of new endosteal bone, partially or entirely bridging the intramedullary canal.

### Quantitative evaluation of bone growth via monochromatic images

**Figure 1:**
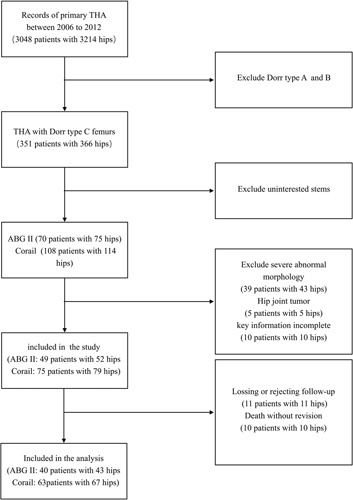
Conventional mixed-energy images showing severe artifacts and monochromatic images obtained with increased photon energy showing fewer artifacts.

To further investigate the difference between short and standard-length stems, representative samples of the two retrospective cohorts (25 from each cohort to lighten the heavy workload) were selected via a random number table and invited to undergo spectral CT examinations at our institution. They were examined on a 64-channel new-generation dual-energy CT scanner (Revolution CT, GE Healthcare) using a dual-energy imaging mode with fast kilovoltage switching between 80 and 140 kVp. The scanning parameters included a beam collimation of 20 mm (32×0.625 mm), a helical pitch of 1.375:1, a tube rotation speed of 0.8 s/turn, and a tube current of 550 mA. The scanning direction was from the head to the foot, and the total exposure time was 5.5 s. Scan data were used to reconstruct monochromatic images with GSI viewer software on a commercial workstation (GE VolumeShare AW4.6; GE Healthcare). Monochromatic energy images were reconstructed at a slice thickness and spacing of 0.625 mm using the bone kernel without applying adaptive statistical iterative reconstruction-V (ASIR-V) or an image enhancement filter. The metal artifact reduction correction was turned off to avoid overcorrection affecting measurement of the space around the stem (Supplementary Fig. 2, Supplemental Digital Content 3, http://links.lww.com/JS9/B181). Monochromatic energy images obtained at 140 kilo-electron volts (keV) of energy were selected for analysis due to their optimal contrast-to-noise ratio and minimal amount of beam hardening artifacts (Fig. 1).

Spot welds in the central coronal and sagittal monochromatic images were counted and summed by two independent authors to evaluate dense bone growth; for trabecular bone growth, the gap around the stems (where the stem did not contact trabecular bone) was measured. In previous studies, gap filling was frequently measured only in animal studies^30,31^ and cadaveric studies^29^ because transverse slices were the most widely used. With recent studies demonstrating the favorable effect of monochromatic images on eliminating metal artifacts from hip prostheses^32,33^, measuring the gap around the stem during follow-up has become possible. The femurs with stems were manually circled out and cut at the conjunction of the neck and shaft. In the femoral canal of the circled part, the gap around the stem was selected by the threshold level. The mean cutoff CT value that separated the gap and the trabecular bone in the first 10 trial patients was calculated to be −615 Hounsfield units (HU); thus, −615 HU was set as the criterion. After automatic extraction, a manual review and fine adjustment (if necessary) were conducted. Subsequently, the volume of the gap was measured with the volume tool equipped in the workstation (Supplementary Fig. 3 shows the workflow, Supplemental Digital Content 4, http://links.lww.com/JS9/B182).

For the quantitative measurement of bone growth in monochromatic images, intraobserver and interobserver intraclass correlation coefficients (ICC) were calculated via the two-way mixed effects, absolute agreement, multiple raters/measurements ICC form^34^ to evaluate the reliability.

### Patient-specific FEA

Patient-specific finite element models, including femurs and stems, were created with Mimics 21.0 (Materialise HQ Technologielaan) and Geomagic Studio 12 (Geomagic, Inc.) software based on preceding monochromatic images. Volume meshes were created using linear tetrahedral elements (C3D4) with a maximal edge length of 3 mm for stems and 2.5 mm for femurs. One hundred material categories were assigned to the femurs based on the HU value. The apparent density (r) was calculated by assuming a linear relationship between the density and HU^35^. Young’s modulus for each material was calculated by the equation proposed by Morgan *et al*.^36^ (E=6850 r ^1.49^). Both the ABG II and Corail are titanium alloy stems, and the corresponding Young modulus was set at 115 GPa. A Poisson ratio of 0.3 was assigned to both bones and implants. The FEA was performed in Abaqus Standard 2018 (Dassault Systemes). The interfacing surfaces between the implant HA coat and femur were set to ʻtiedʼ. Loading with hip contact and muscle forces in level walking was applied in each model. Action points, directions, and strengths of different muscles were set by referring to a study by Heller *et al*.^37^. The most distal 2 cm of the femur was set as a constrained boundary. To explore the risk of proximal fracture, the mean principle strain of the 10 elements with maximum values in the proximal femur (5 cm below the lower border of the lesser trochanter^38^) were calculated and compared^39^.

### Statistical analysis

Student’s *t*-test was used for continuous variables, and Fisher’s exact test or the *χ*^2^ test was applied for categorical variables. The cumulative survival rate for stems with revision as the endpoint was calculated by Kaplan–Meier survivorship analysis. Multivariate Cox regression analysis was performed, and adjusted hazard ratios (HRs) for different predictors of revision were calculated. Statistical analysis was conducted with SPSS software (version 25.0; SPSS Science). A two-tailed *P*-value of <0.05 was considered significant. Intraobserver and interobserver ICCs were calculated to evaluate the reliability of the newly developed measurements, with ICC >0.75 (or >90) indicating good (or excellent) reliability.

## Results

**Figure 2:**
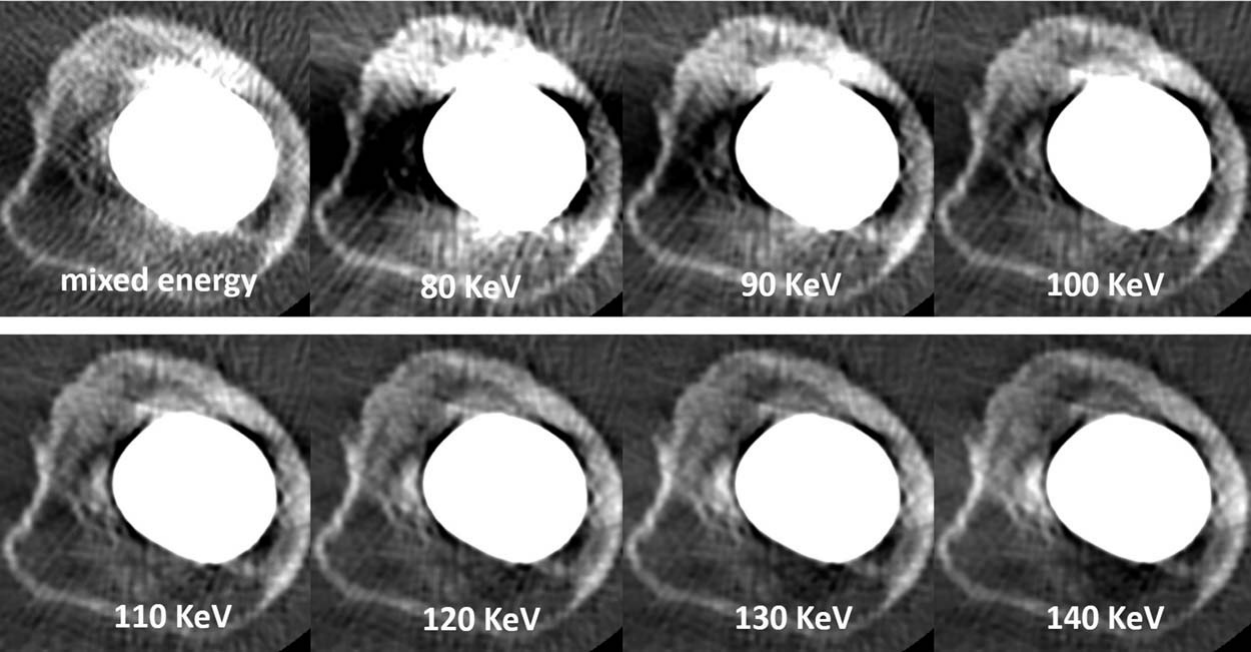
Flowchart showing the inclusion of patients in this study.

Among the 3214 primary THAs, 366 THAs in 351 patients were conducted in Dorr type C femurs, and 189 THAs involved the stems of interest, that is, the ABG II or Corail stem. Among the 124 identified patients who underwent 131 eligible THAs, 11 patients (11 THAs) were lost to follow-up or rejected, and another 10 patients (10 THAs) died without revision, leaving 103 patients with 110 THAs (43 ABG II stems and 67 Corail stems) in this retrospective cohort study (Fig. 2). All included patients underwent THA under general anesthesia with a posterior-lateral approach. There was no significant difference in the mean age, weight, height, BMI, preoperative Harris hip score (HHS), operating time, sex ratio, comorbidity, or diagnosis between the two groups (Table 1).

**Table 1:** Patients demographics at the surgery.

Demographics	ABG II group	Corail group	*P*
Number of patients (hips)	40 (43)	63 (67)	—
Sex, male: female	16: 24	31: 32	0.84
Mean age (range)	57.7 (38–82)	54.1 (19–84)	0.22
Mean weight, kg (range)	59.8 (40–85)	57.1 (38–80)	0.26
Mean height, cm (range)	160.0 (144–173)	160.3 (135–178)	0.90
Mean BMI, kg/m^2^ (range)	23.1 (15.6–28.7)	22.2 (14.7–30.3)	0.13
Diagnosis	DDH: 7 hips Femoral necrosis: 9 hips Femoral neck fracture: 12 hips Osteoarthritis: 7 hips Ankylosing Spondylitis: 4 hips Others: 4 hips	DDH: 14 hips Femoral necrosis: 13 hips Femoral neck fracture: 8 hips Osteoarthritis: 7 hips Ankylosing Spondylitis: 9 hips Others: 12 hips	0.20
Number with Comorbidity (percentage)	12 (30.0%)	24 (38.1%)	0.40
Preoperative Harris score (range)	38.0 (21–73)	34.8 (20–64)	0.20
Operation time, min (range)	118.0 (60–190)	114.5 (45–270)	0.65
Average follow-up^a^, months (range)	145.7 (119–163)	109.0 (91–167)	<0.05^b^

a Patients with revisions did not count.

b In previous studies^53,54^, the survival rate of Corail stems decreased by less than 1% from the 9th to the 12th year.

### Revision rates and radiologic signs

**Figure 3:**
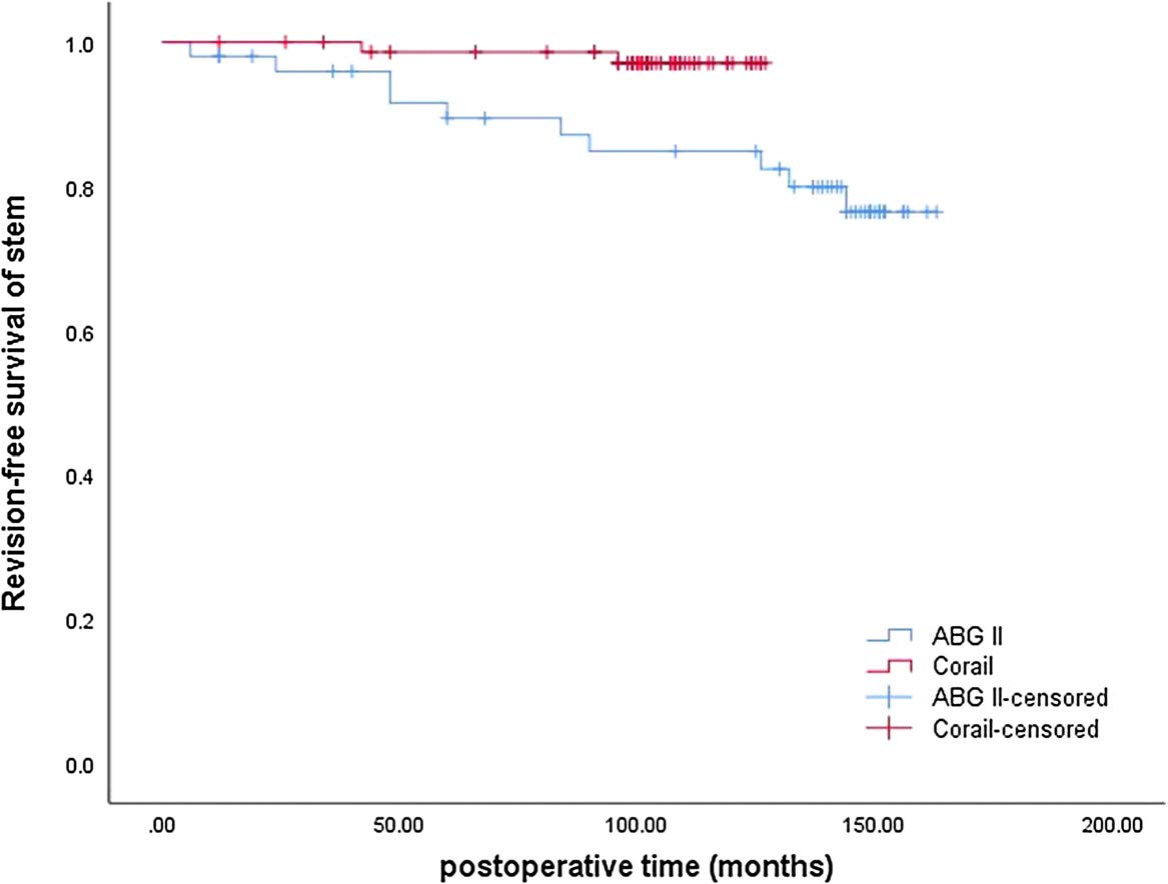
Kaplan–Meier survival curve showing better survival in the Corail group.

The revision rate of stems in the ABG II group was significantly higher than that of stems in the Corail group (21 vs. 3%, *P*<0.05). Revision was performed for one periprosthetic joint infection (2%) and 8 postoperative PFFs (19%) in the ABG II group and 1 (1%) periprosthetic joint infection and 1 postoperative PFF (1%) in the Corail group (Table 2). The Kaplan–Meier curve (Fig. 3) presented a survival rate of 76.4% in the ABG II group at 143 months (95% CI: 132.2–155.7 months) versus a survival rate of 97.1% in the Corail group at 125 months (95% CI: 122.9–127.8 months). A significant difference between the two groups was shown by the log-rank test (*P*=0.013). After adjusting for age, sex, BMI, preoperative HHS, time of surgery, fill ratio, diagnosis, and intraoperative fracture in the Cox regression, the hazard ratio of ABG II stems was 13.4 (*P*<0.05). The Kaplan–Meier curve trends also indicated that the survival difference was not caused by the longer follow-up time in the ABG II group. Comparison of the HHS, satisfaction score, and radiology signs showed no significant difference between the two groups, except that more pedestal signs were present in the Corail group than in the ABG II group (56.9 vs. 14.7%, *P*<0.001) (Table 2).

**Table 2:** Clinical and radiological outcomes of all included patients.

Parameters	ABG II group	Corail group	*P*
Both patients with revision or without revision			
Number of stems	43	67	—
Postoperative fracture (*n*, %)	10, 23%	3, 4%	0.009
Overall revision of stem (*n*, %)	9, 21%	2, 3%	0.010
Patients without revision (results at 10.3-year follow-up)			
Number of stems	34	65	—
Mean HHS (SD; range)	85.0 (19.3; 10–99)	89.6 (19.3; 4–99)	0.26
Satisfaction score (SD; range)	87.2 (12.8; 50–100)	91.3 (10.7; 40–100)	0.09
Stability (osseous fixation: fibrous fixation)	33:1	64:1	0.64
Spot welds	23 (67.6%)	50 (76.9%)	0.32
Radiolucent lines	6 (17.6%)	4 (6.2%)	0.08
Heterotopic ossification	1 (3.0%)	0 (0%)	0.28
Pedestals	5 (14.7%)	37 (56.9%)	<0.001
Osteolysis	1 (3.0%)	0 (0%)	0.28
Stress shielding (none or first degree: second degree)	15: 19	35:30	0.36

### Bone growth

The reliability of measuring bone growth in monochromatic images is good to excellent (spot welds, ICC=0.89 for intraobserver and 0.86 for interobserver; gap volume, ICC=0.97 for intraobserver and 0.95 for interobserver).

**Figure 4:**
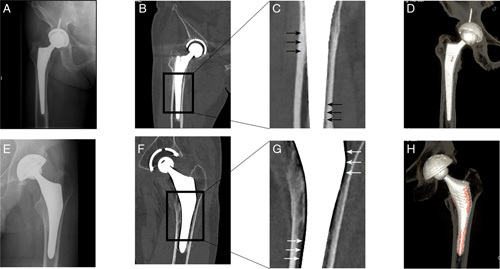
Corail stems presented better bone growth than ABG II stems in monochromatic images. A and E: Plain X-rays showing no radiolucent lines or spot welds. B and C: Monochromatic images of Corail stems showing obvious spot welds (black arrow). F and G: Monochromatic images showing radiolucent lines (white arrow), that is, the gap, around the ABG II stem. D and H: 3D images showing much larger gaps (colored structure) around the ABG II stems than around the Corail stems.

The central coronal and sagittal monochromatic images showed fewer spot-weld signs in the ABG II group than in the Corail group (2.2 in the ABG II group and 3.4 in the Corail group on average, *P*<0.001). The mean gap volume around the stem calculated via spectral CT in the ABG II group (1.64 cm^3^±1.28 cm^3^) was much larger than that in the Corail group (0.13 cm^3^±0.19 cm^3^) (*P*<0.001). Even accounting for the difference in the mean volume of the two stems embedded in the femurs (32.6 cm^3^ for ABG II vs. 23.0 cm^3^ for Corail), the gap-to-stem volume ratio in the ABG II group was still much larger than that in the Corail group. The 3D images showed striking gaps around both the proximal and distal parts of the ABG II stems, which differed from the Corail stems (Figs 4D and H). Notably, spot welds and radiolucent lines might be invisible in plain X-ray images but prominent in monochromatic images (Figs 4A, B, C, E, F, and G).

### Femoral strains

**Figure 5:**
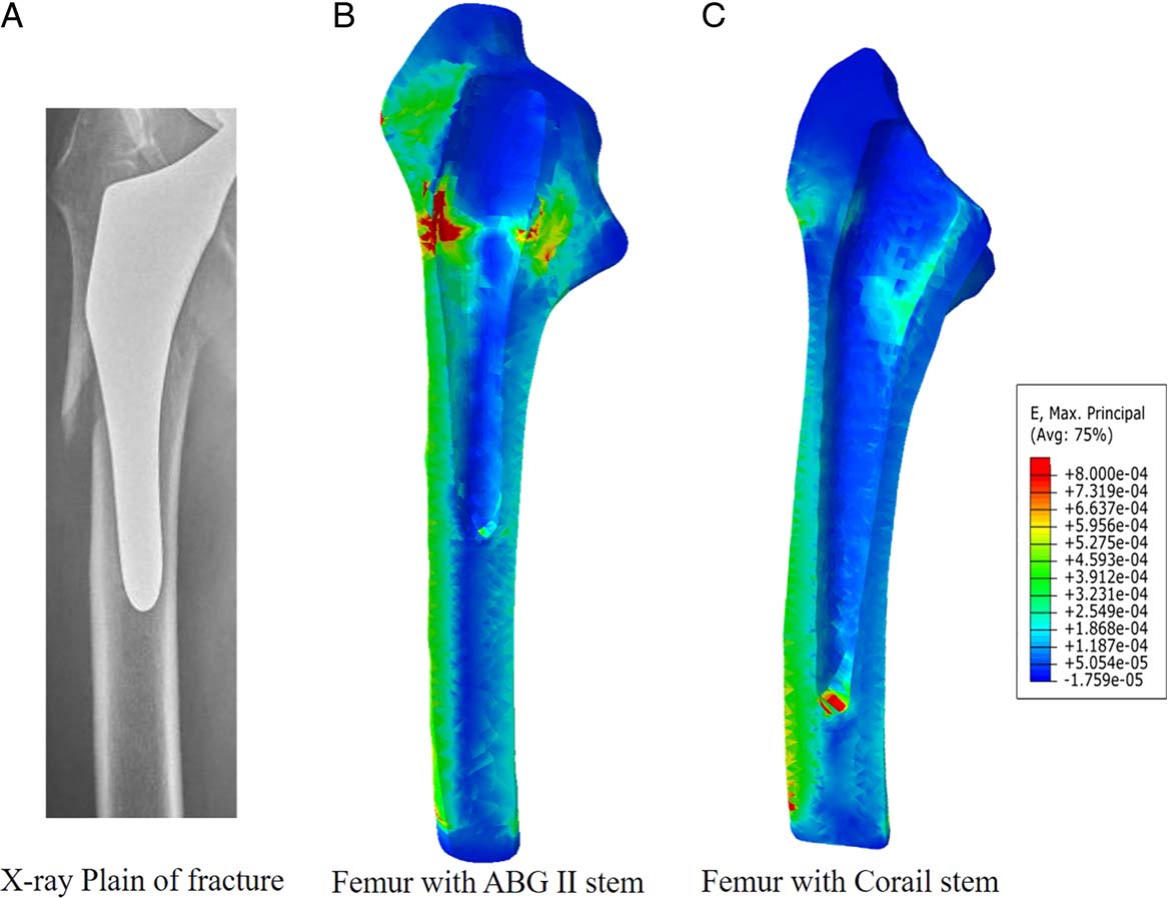
PFFs and high proximal femoral strains were present in patients who received ABG II stems. A: X-ray film showing proximal PFFs in a patient in the ABG II group in the sixth year after surgery. B: A typical strain nephogram of the ABG II stem showing the maximum principal strain (red color) present in the proximal femur. C: Typical strain nephogram of the Corail stem showing the maximum principal strain (red color) in the distal femur.

The mean maximum principal strains in the proximal femur of the 25 patients in the ABG II group (0.0052±0.0020) were significantly larger than those in the Corail group (0.0011±0.0006), with a *P*-value <0.001. This difference might result from the different distribution of strain in the femur with the two stems in the ABG II group, most patients (21/25) showed the highest strains in the proximal part, while in the Corail group, most patients (18/25) showed the highest strains in the distal part. A typical femoral strain distribution is shown in the strain nephogram in Figure 5.

## Discussion

Little is known about the long-term results of short cementless stems in Dorr type C femurs, and no long-term results of short stems in patients with Dorr type C femurs have been published yet. Therefore, we followed patients with Dorr type C femurs who underwent THA with ABG II stems and Corail stems and compared them to evaluate the long-term performance. The main findings of this study are that there is a significantly higher risk of postoperative PFFs and revision for ABG II stems used in Dorr type C femurs. To further investigate the potential cause of PFFs in the ABG II group, spectral CT and patient-specific FEA were conducted. Poorer bone growth and higher proximal strains were detected in the ABG II group, which might be potential causes of the unsatisfactory performance.

We systematically reviewed the revision rate of stems in studies focused on Dorr type C femurs^7,15,18,22,24,40–46^ (Table 3). The survival rate of Corail stems in our study (97%) is consistent with that in previous studies on other standard-length stems in Dorr type C femurs (93 to 100%). For short stems in Dorr type C femurs, only short-term outcomes have been reported in small case series. Liu *et al*.^7^ and Patel *et al*.^8,9^ reported satisfactory results of short stems in Dorr type C femurs, while Gkagkalis *et al*.^14^ applied short stems (Optimys) in nine Dorr type C femurs, discovering two postoperative PFFs in a mean 49-month prospective follow-up. In this study, long-term results were revealed for the first time, and a high postoperative PFF risk was detected. Interestingly, without being limited to Dorr type C femurs, ABG II stems have been reported to have a survival rate of over 98% after an average of 10 years^12,13^, and Epinette *et al*. reported no significant difference in 8-year survival between ABG II stems and standard-length stems (Omnifit). It seems that the survival of ABG II stems is altered dramatically when the type of femur changes and that the risk of short stems in Dorr type C femurs is obviously higher than that in general femurs.

**Table 3:** Systematic review of studies that focused on cementless stems in Dorr type C femurs.

Author, year	Hips	Follow-up	Stems	HHS	Thigh pain	Fixation on X-Ray	Revision
Keisu, 2001^43^	23	5 years	Taperloc (Zimmer Biomet, US)	—	—	All bone ingrowth	0
Reitman, 2003^18^	33	13.2 years	Mallory-Head press-fit (Biomet, US).	—	—	—	0
Kelly, 2007^19^	5	11.5 years	Omnifit HA stem (Stryker, US)	94.5	1	All bone ingrowth	0
Meding, 2010^42^	127	5.9 years	Bi-Metric (Biomet, US)	94.7	—	—	0
Dalury, 2012^24^	43	6 years	Summit (Depuy Orthopedics, US)	94	0	All bone ingrowth	0
Hatem, 2014^40^	18	2.8 years	Bicontact model (Aesculap, Germany)	86	—	—	0
Takigam, 2013	7	9.4 years	SL-PLUS (Smith & Nephew Orthopedics, US)	—	—	—	0
Bonutti^a^, 2014^41^	105	6 years	Secure-Fit (Stryker, US)	80	—	—	8
Kang^a^, 2015^45^	41	3.8 years	Wagner Cone (Zimmer, US)	87.9	—	—	0
Mclaughlin, 2016^20^	60	16.6 years	Taperloc (Zimmer Biomet, US)	89	3	56 bone ingrowth; 3 fibrous ingrowth	1
Tsubosaka, 2018^44^	17	5.5 years	Summit cementless stem(Depuy, US)	81.0	—	—	0
Liu, 2019^7^	37	8 months	Tri-Lock^b^ (DePuy Orthopedics, US.)	93.3	0	—	0
Zhen, 2019^21^	28	10.4 years	Wagner self-locking (Zimmer, US)	90	0	23 bone ingrowth; two fibrous fixation.	0
Kim, 2019^22^	62	13.6 years	Bicontact (AESCULAP AG and Co, Germany)	96.5	0	—	0
Zhen, 2021^15^	42	5.5 years	Tri-Lock^b^ (DePuy Orthopedics, US.)	91.0	0	All bone ingrowth	0

a For hemiarthroplasty.

b For short stem.

Our results demonstrated poorer bone growth with ABG II stems than Corail stems in Dorr type C femurs in terms of both dense bone (number of spot welds) and trabecular bone (inverse of gap volume around stems). In Dorr type C femurs, the ʻstovepipeʼ morphology adds extra difficulty in initial fixation, and cellular abnormalities^1^ create a less favorable environment for bone ingrowth. We speculate that the potential distal fixation, wider and thicker HA coating, and subsidence-resistant double-tapered design, which are not features provided by ABG II stems, make Corail stems better for bone growth. Osteoporosis might also affect bone growth, and the percentage of patients diagnosed with osteoporosis in the ABG II group was even lower than that in the Corail group, according to the medical records, demonstrating that the effect of the stems might be more prominent.

In addition to poor fixation due to less bone growth around ABG II stems in Dorr type C femurs, the results indicating high proximal femoral strain might also account for the PFFs. Previous cadaveric biomechanical studies presented contradictory results regarding fracture and stem length. Jakubowitz *et al*.^47^ reported that short stems do not confer a higher fracture risk, while Klasen *et al*.^48^ claimed that the primary load at failure in short stems was significantly lower than that in standard-length stems. These contradictions might be caused by femoral variability, which is of enormous importance in the results^49^; therefore, patient-specific analysis in Dorr type C femurs is necessary. In our patient-specific FEA, the mean maximum principal strains of the proximal femur in the ABG II group (0.0052) were much higher than those in the Corail group (0.0011), considering that the yield tensile strain for trabecular bone and cortical bone in the research reported by Bayraktar *et al*.^50^ was 0.0062 and 0.0073, respectively. We speculated that proximal fixation and the ultra-polished distal part of the ABG II stem caused larger strains in the metaphysis, while the tip of the fully coated and standard-length Corail stem frequently contacted the diaphysis or connected to the diaphysis by a pedestal, reducing the strain in the proximal part. A previous study reported that distal contact could reduce proximal strain, leading to proximal bone resorption, which was considered a shortcoming of standard-length stems^51^. However, in our research, when Dorr type C femurs were the focus, the maximum principal strains of proximal femurs with ABG II stems were near the yield tensile strains for bone, and potential distal contact inversely became a safer option.

The main significance of this study lies in two aspects. On the one hand, it revealed a high and long-term risk of PFF for ABG II stems in Dorr type C femurs for the first time, which places an emphasis on femoral morphology and partially explains the contradiction of some researchers reporting a high revision rate for ABG II stems^52,53^ and others reporting good survival^12,13^. On the other hand, it provided some evidence that poor bone ingrowth and high proximal strains might cause failure of the femoral stem, which could draw attention to other short stems in Dorr type C femurs.

This study had several limitations. First, this study was conducted retrospectively; we introduced quantitative measurements and discovered poor bone growth and high proximal femoral strains with ABG II stems in Dorr type C femurs, which were speculated to be the causes of failure. However, a prospective study is necessary to verify this causality. Second, we recognize the potential bias of this retrospective cohort study. The choice of stem depended on the preference of the surgeon, which might introduce confounding factors. One direct result was that the ABG II group had a smaller sample size because the surgeons began to reduce the application of ABG II stems after some revisions were needed. Additionally, the surgical procedure for the two types of stems is not identical, and the three surgeons might have different operating preferences. Although each of the three senior surgeons performed more than 100 THAs per year, some surgical details might also cause bias. Third, no previous studies have quantitively investigated the volume of the gap around the stem; therefore, the standard for measuring the gap depends on similar studies and our own experience, and the reliability of this measurement needs to be validated in vitro after the stems are revised and extracted. Further research to optimize this procedure should be conducted. Fourth, the mean follow-up in the ABG II group was significantly longer than that in the Corail group (12.1 year vs. 9.1 year, *P*<0.001) (Table 1). In previous studies^54,55^, the survival rate of Corail stems decreased by less than 1% from the 9th to the 12th year. Therefore, the difference in follow-up probably had a minor effect on the results. Fifth, we only examined ABG II stems and Corail stems, and the results cannot be extrapolated to other stems. Studies on other short stems in Dorr type C femurs are still necessary.

## Conclusions

There was a high risk of postoperative PFF and revision for ABG II stems in Dorr type C femurs. Quantitative detection of poor bone growth and high proximal strains provided insight into the failure mechanism of short stems in Dorr type C femurs. Based on monochromatic images, researchers might make new discoveries and gain more understanding of joint prostheses in future studies.

## Ethical approval

This study was approved by the ethics committee of the West China Hospital, and written consent was obtained from all patients. Each author certifies that his institution approved the human protocol for this investigation and that all investigations were conducted in conformity with ethical principles of research.

## Consent

Written informed consent was obtained from the patient for publication and any accompanying images. A copy of the written consent is available for review by the Editor-in-Chief of this journal on request.

## Sources of funding

One of the authors (S.B.) has received, during the study period, funding from the Foundation of the Science and Technology Department of Sichuan Province (2022YFS0050). One of the authors (L.M.Y.) has received, during the study period, funding from the Foundation of the Science and Technology Department of Sichuan Province (2023NSFSC1750) and Sichuan University Postdoctoral Interdisciplinary Innovation Fund (JCXK2217).

## Author contribution

M.L., Y.Z., Y.N., and B.S.: concept/idea/research design; M.L. and Y.Z.: acquisition of data; Y.Z. and Y.N.: analysis and interpretation of data; M.L. and H.X.: writing/review/editing/revising of manuscript; B.S.: final approval of the manuscript; B.S. and M.L.: acquisition of funding; K.L., F.P., and J.Y.: providing facilities/equipment; F.P., J.Y., B.S.: providing subjects.

## Conflicts of interest disclosure

All authors declare no conflicts of interest.

## Research registration unique identifying number (UIN)

The study was registered in the Chinese Clinical Trial Registry (ChiCTR 1900026467). chictr.org.cn/showprojEN.html?proj=44138.

## Guarantor

Shen bin accept full responsibility for the work and/or the conduct of the study, had access to the data, and controlled the decision to publish.

## Data availability statement

Data are available upon request to the corresponding author.

## Provenance and peer review

Not commissioned, externally peer-reviewed.

## References

[1] Dorr LD Faugere M-C Mackel AM . Structural and cellular assessment of bone quality of proximal femur. Bone 1993;14:231–242. 8363862 10.1016/8756-3282(93)90146-2

[2] Christie M Brinson MF . Proximal/distal mismatch: type A and C femurs. Orthopedics 2005;28(Suppl 9):s1033–s1036. 16190030 10.3928/0147-7447-20050902-05

[3] Gromov K Bersang A Nielsen C . Risk factors for post-operative periprosthetic fractures following primary total hip arthroplasty with a proximally coated double-tapered cementless femoral component. Bone Joint J 2017;99:451–457. 28385933 10.1302/0301-620X.99B4.BJJ-2016-0266.R2

[4] Molli RG Lombardi AV Berend KR . A short tapered stem reduces intraoperative complications in primary total hip arthroplasty. Clin Orthop Rel Res 2012;470:450–461. 10.1007/s11999-011-2068-7 PMC3254753 21971877

[5] Kim Y-H Park J-W Kim J-S . Long-term results and bone remodeling after THA with a short, metaphyseal-fitting anatomic cementless stem. Clin Orthop Rel Res 2014;472:943–950. 10.1007/s11999-013-3354-3 PMC3916612 24163094

[6] Chen HH Morrey BF An KN . Bone remodeling characteristics of a short-stemmed total hip replacement. J Arthroplasty 2009;24:945–950. 18848420 10.1016/j.arth.2008.07.014

[7] Liu J Qu T Li XS . Application of bone-retaining femoral stem prosthesis in young patients with Dorr C femoral medullary cavity. [Chinese] Zhongguo gu shang China J Orthop Traumatol 2019;32:785–791. 10.3969/j.issn.1003-0034.2019.09.002 31615171

[8] Patel RM Smith MC Woodward CC . Stable fixation of short-stem femoral implants in patients 70 years and older. Clin Orthop Rel Res 2012;470:442–449. 10.1007/s11999-011-2063-z PMC3254743 21927967

[9] Patel R Lo W Cayo M . Stable, Dependable Fixation of Short-stem Femoral Implants at 5 Years. Orthopedics 2013;36:e301–e307. 23464949 10.3928/01477447-20130222-18

[10] Aro E Alm JJ Moritz N . Good stability of a cementless, anatomically designed femoral stem in aging women: a 9-year RSA study of 32 patients. Acta Orthop 2018;89:490–495. 29987941 10.1080/17453674.2018.1490985 PMC6202764

[11] Issa K Pivec R Wuestemann T . Radiographic fit and fill analysis of a new second-generation proximally coated cementless stem compared to its predicate design. J Arthroplasty 2014;29:192–198. 23706811 10.1016/j.arth.2013.04.029

[12] Epinette JA Asencio G Essig J . Clinical results, radiological findings and survival of a proximally hydroxyapatite-coated hip ABG II stem at a minimum of ten years’ follow-up: results of a consecutive multicentre study of 1148 hips in 1053 patients. Bone Joint J 2013;95-b:1610–1616. 24293589 10.1302/0301-620X.95B12.31167

[13] Herrera A Mateo J Lobo-Escolar A . Long-term outcomes of a new model of anatomical hydroxyapatite-coated hip prosthesis. J Arthroplasty 2013;28:1160–1166. 23134598 10.1016/j.arth.2012.06.033

[14] Gkagkalis G Goetti P Mai S . Cementless short-stem total hip arthroplasty in the elderly patient - is it a safe option?: a prospective multicentre observational study. BMC Geriatr 2019;19:112. 30995903 10.1186/s12877-019-1123-1 PMC6472082

[15] Zhen P Chang Y Yue H . Primary total hip arthroplasty using a short bone-conserving stem in young adult osteoporotic patients with Dorr type C femoral bone. J Orthop Surg 2021;16:17. 10.1186/s13018-020-01985-z PMC7789779 33413495

[16] Mathew G Agha R Albrecht J . STROCSS 2021: strengthening the reporting of cohort, cross-sectional and case-control studies in surgery. Int J Surg 2021;96:106165. 34774726 10.1016/j.ijsu.2021.106165

[17] Chow S-C Shao J Wang H . Sample size calculations in clinical research. CRC press; 2017.

[18] Reitman RD Emerson R Higgins L . Thirteen year results of total hip arthroplasty using a tapered titanium femoral component inserted without cement in patients with type C bone. J Arthroplasty 2003;18(7 Suppl 1):116–121. 14560420 10.1016/s0883-5403(03)00344-9

[19] Kelly SJ Robbins CE Bierbaum BE . Use of a hydroxyapatite-coated stem in patients with Dorr Type C femoral bone. Clin Orthop Relat Res 2007;465:112–116. 17704696 10.1097/BLO.0b013e318156bf96

[20] McLaughlin JR Lee KR . Long-term results of uncemented total hip arthroplasty with the Taperloc femoral component in patients with Dorr type C proximal femoral morphology. Bone Joint J 2016;98-b:595–600. 27143728 10.1302/0301-620X.98B5.35816

[21] Zhen P Liu J Li X . Primary total hip arthroplasty using an uncemented Wagner SL stem in elderly patients with Dorr type C femoral bone. J Orthop Surg 2019;14:377. 10.1186/s13018-019-1421-5 PMC6868742 31752915

[22] Kim JT Jeong HJ Lee SJ . Does proximally coated single-wedge cementless stem work well in dorr type C Femurs? Minimum 10-year follow-up. Indian J Orthop 2019;53:94–101. 30905988 10.4103/ortho.IJOrtho_160_17 PMC6394166

[23] Park C-W Eun H-J Oh S-H . Femoral stem survivorship in dorr type a femurs after total hip arthroplasty using a cementless tapered wedge stem: a matched comparative study with type B Femurs. J Arthroplasty 2019;34:527–33. 30545654 10.1016/j.arth.2018.11.004

[24] Dalury DF Kelley TC Adams MJ . Modern proximally tapered uncemented stems can be safely used in dorr type c femoral bone. J Arthropl 2012;27:1014–1018. 10.1016/j.arth.2011.12.019 22325961

[25] Nakaya R Takao M Hamada H . Reproducibility of the Dorr classification and its quantitative indices on plain radiographs. Orthop Traumatol Surg Res 2019;105:17–21. 30594598 10.1016/j.otsr.2018.11.008

[26] Feyen H Shimmin AJ . Is the length of the femoral component important in primary total hip replacement? Bone Joint J 2014;96-b:442–448. 24692608 10.1302/0301-620X.96B4.33036

[27] Harris WH . Traumatic arthritis of the hip after dislocation and acetabular fractures: treatment by mold arthroplasty. An end-result study using a new method of result evaluation. J Bone Joint Surg Am 1969;51:737–755. 5783851

[28] Engh CA Massin P Suthers KE . Roentgenographic assessment of the biologic fixation of porous-surfaced femoral components. Clin Orthop Relat Res 1990:107–128. 2199114

[29] Engh CA Bobyn J Glassman AH . Porous-coated hip replacement. The factors governing bone ingrowth, stress shielding, and clinical results. J Bone Joint Surg Br 1987;69:45–55. 3818732 10.1302/0301-620X.69B1.3818732

[30] Dalton JE Cook SD Thomas KA . The effect of operative fit and hydroxyapatite coating on the mechanical and biological response to porous implants. JBJS 1995;77:97–110. 10.2106/00004623-199501000-00012 7822360

[31] Sandborn PM Cook SD Spires WP . Tissue response to porous-coated implants lacking initial bone apposition. J Arthroplasty 1988;3:337–346. 3241171 10.1016/s0883-5403(88)80034-2

[32] Magarelli N De Santis V Marziali G . Application and advantages of monoenergetic reconstruction images for the reduction of metallic artifacts using dual-energy CT in knee and hip prostheses. Radiol Med (Torino) 2018;123:593–600. 29637389 10.1007/s11547-018-0881-8

[33] Wang F Xue H Yang X . Reduction of metal artifacts from alloy hip prostheses in computer tomography. J Comput Assist Tomogr 2014;38:828–833. 24983437 10.1097/RCT.0000000000000125

[34] McGraw KO Wong SP . Forming inferences about some intraclass correlation coefficients. Psychol Methods 1996;1:30.

[35] Bitsakos C Kerner J Fisher I . The effect of muscle loading on the simulation of bone remodelling in the proximal femur. J Biomech 2005;38:133–139. 15519348 10.1016/j.jbiomech.2004.03.005

[36] Morgan EF Bayraktar HH Keaveny TM . Trabecular bone modulus-density relationships depend on anatomic site. J Biomech 2003;36:897–904. 12757797 10.1016/s0021-9290(03)00071-x

[37] Heller MO Bergmann G Kassi JP . Determination of muscle loading at the hip joint for use in pre-clinical testing. J Biomech 2005;38:1155–1163. 15797596 10.1016/j.jbiomech.2004.05.022

[38] Egol KA Leucht P . Proximal femur fractures: an evidence-based approach to evaluation and management. Springer; 2017.

[39] Taylor M Prendergast PJ . Four decades of finite element analysis of orthopaedic devices: where are we now and what are the opportunities? J Biomech 2015;48:767–778. 25560273 10.1016/j.jbiomech.2014.12.019

[40] Ahmad Hatem M Ferreira da Luz B Nishimoto Nishi R . Evaluation of the results from proximal fixation of uncemented conical femoral components in Dorr type C femurs. Rev Bras Ortop 2014;49:260–266. 26229810 10.1016/j.rboe.2014.02.007 PMC4511651

[41] Bonutti PM Stroh AD Issa K . Proximally coated cementless bipolar hemiarthroplasty in Dorr type C bone. Orthopedics 2014;37:e345–e350. 24762838 10.3928/01477447-20140401-54

[42] Meding JB Galley MR Ritter MA . High survival of uncemented proximally porous-coated titanium alloy femoral stems in osteoporotic bone. Clin Orthop Relat Res 2010;468:441–447. 19727996 10.1007/s11999-009-1035-z PMC2806973

[43] Keisu KS Orozco F Sharkey PF . Primary cementless total hip arthroplasty in octogenarians. Two to eleven-year follow-up. J Bone Joint Surg Am 2001;83:359–363. 11263639 10.2106/00004623-200103000-00007

[44] Tsubosaka M Hayashi S Hashimoto S . Patients with a Dorr type C femoral bone require attention for using a Summit cementless stem: results of total hip arthroplasty after a minimum follow-up period of 5 years after insertion of a Summit cementless stem. J Orthop Sci 2018;23:671–675. 29853316 10.1016/j.jos.2018.05.001

[45] Kang JH Lee SH Jung S . Bipolar hemarthroplasty using cementless conical stem for treatment of Dorr Type B and C femoral neck fracture. Hip Pelvis 2015;27:232–240. 27536631 10.5371/hp.2015.27.4.232 PMC4972794

[46] Takigami I Ito Y Matsumoto K . Mid-Term results of the SL-PLUS femoral prosthesis the influence of femoral bone type. Bull Hosp Jt Dis 2017;75:128–133. 28583059

[47] Jakubowitz E Seeger JB Lee C . Do short-stemmed-prostheses induce periprosthetic fractures earlier than standard hip stems? A biomechanical ex-vivo study of two different stem designs. Arch Orthop Trauma Surg 2009;129:849–855. 18568351 10.1007/s00402-008-0676-9

[48] Klasan A Bäumlein M Dworschak P . Short stems have lower load at failure than double-wedged stems in a cadaveric cementless fracture model. Bone Joint Res 2019;8:472–80. 10.1302/2046-3758.810.BJR-2019-0051.R1 PMC6825045 31728187

[49] Dopico-González C New AM Browne M . Probabilistic finite element analysis of the uncemented hip replacement—effect of femur characteristics and implant design geometry. J Biomech 2010;43:512–520. 19896129 10.1016/j.jbiomech.2009.09.039

[50] Bayraktar HH Morgan EF Niebur GL . Comparison of the elastic and yield properties of human femoral trabecular and cortical bone tissue. J Biomech 2004;37:27–35. 14672565 10.1016/s0021-9290(03)00257-4

[51] Kim YH Kim JS Cho SH . Strain distribution in the proximal human femur. An in vitro comparison in the intact femur and after insertion of reference and experimental femoral stems. J Bone Joint Surg Br 2001;83:295–301. 11284584 10.1302/0301-620x.83b2.10108

[52] Thien TM Chatziagorou G Garellick G . Periprosthetic femoral fracture within two years after total hip replacement: analysis of 437,629 operations in the nordic arthroplasty register association database. J Bone Joint Surg Am 2014;96:e167. 25274795 10.2106/JBJS.M.00643

[53] Mulford JS Mathew R Penn D . Periprosthetic fracture as a late mode of failure of the Anatomique Benoist Girard II femoral prosthesis. ANZ J Surg 2022;92:1165–70. 35191171 10.1111/ans.17547 PMC9306843

[54] Vidalain J-P . Twenty-year results of the cementless Corail stem. Int Orthop 2011;35:189–194. 20814676 10.1007/s00264-010-1117-2 PMC3032112

[55] Louboutin L Viste A Desmarchelier R . Long-term survivorship of the Corail™ standard stem. Orthop Traumatol 2017;103:987–92. 10.1016/j.otsr.2017.06.010 28778624

